# Clinical evaluation of an anatomy‐based patient specific quality assurance system

**DOI:** 10.1120/jacmp.v15i2.4647

**Published:** 2014-03-06

**Authors:** Pascal Hauri, Sarah Verlaan, Shaun Graydon, Linda Ahnen, Stephan Klöck, Stephanie Lang

**Affiliations:** ^1^ Department of Radiation Oncology University Hospital Zurich Zurich Switzerland

**Keywords:** patient‐specific quality assurance, volumetric‐modulated arc therapy, DVH‐based QA

## Abstract

The Delta^4DVH^ Anatomy 3D quality assurance (QA) system (ScandiDos), which converts the measured detector dose into the dose distribution in the patient geometry was evaluated. It allows a direct comparison of the calculated 3D dose with the measured back‐projected dose. In total, 16 static and 16 volumetric‐modulated arc therapy (VMAT) fields were planned using four different energies. Isocenter dose was measured with a pinpoint chamber in homogeneous phantoms to investigate the dose prediction by the Delta^4DVH^ Anatomy algorithm for static fields. Dose distributions of VMAT fields were measured using GAFCHROMIC film. Gravitational gantry errors up to 10° were introduced into all VMAT plans to study the potential of detecting errors. Additionally, 20 clinical treatment plans were verified. For static fields, the Delta^4DVH^ Anatomy predicted the isocenter dose accurately, with a deviation to the measured phantom dose of 1.1%±0.6%. For VMAT fields the predicted Delta^4DVH^ Anatomy dose in the isocenter plane corresponded to the measured dose in the phantom, with an average gamma agreement index (GAI) (3 mm/3%) of 96.9±0.4%. The Delta^4DVH^ Anatomy detected the induced systematic gantry error of 10° with a relative GAI (3 mm/3%) change of 5.8%±1.6%. The conventional Delta^4PT^ QA system detected a GAI change of 4.2%±2.0%. The conventional Delta^4PT^ GAI (3 mm/3%) was 99.8%±0.4% for the clinical treatment plans. The mean body and PTV‐GAI (3 mm/5%) for the Delta^4DVH^ Anatomy were 96.4%±2.0% and 97.7%±1.8%; however, this dropped to 90.8%±3.4% and 87.1%±4.1% for passing criteria of 3 mm/3%. The anatomy‐based patient specific quality assurance system predicts the dose distribution correctly for a homogeneous case. The limiting factor for the error detection is the large variability in the error‐free plans. The dose calculation algorithm is inferior to that used in the TPS (Eclipse).

PACS numbers: 87.56.Fc, 87.56.‐v

## I. INTRODUCTION

**Figure 1:**
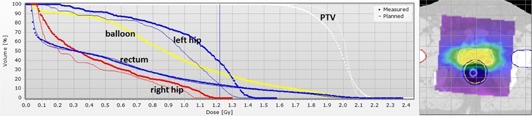
DVHs of the PTV and OAR for a prostate cancer patient. The thin lines are the TPS calculated and the large round points are the measured reprojected Delta^4DVH^ Anatomy DVHs.

Patient‐specific quality assurance (QA) for intensity‐modulated techniques is often performed by comparing measured with calculated dose distributions in a QA phantom. The patient plan is calculated on the QA phantom in the treatment planning system (TPS) and then irradiated. A gamma agreement index (GAI, percentage of point passing the criteria)^(1)^ for the plan is calculated using different acceptance criteria for the dose difference (DD) and distance to agreement (DTA).^(1)^ This method, using the conventional Delta^4PT^ (ScandiDos, Uppsala, Sweden) device, has been proven to be accurate and reproducible.^2^, ^3^, ^4^ However, there is a lack of correlation between conventional QA and dose errors in the patient anatomy.^(5)^ The points, which do not pass the acceptance criteria, can be viewed in the Delta^4PT^ detector array, but nothing can be concluded on the clinical relevance of these points. A solution might be a patient anatomy‐based QA system. The principle of such a system is to measure the dose of the patient plan as for conventional QA in a phantom, and then remap the measured dose distribution to the CT dataset of the patient. This allows a direct comparison of the planned and the measured back‐projected, three‐dimensional (3D) dose to the patient. An advantage compared to conventional QA is that the GAI can be individually evaluated based on the patient body, the planning target volume (PTV), and organs at risk (OAR). Clinically relevant dose comparisons can be achieved. Additionally, organ specific dose‐volume histograms (DVH) can be compared with the TPS predicted DVHs (Fig. 1). This allows discussion regarding potentially necessary clinical plan adjustments with the clinicians, in case of poor agreement in the patient's DVH Anatomy analysis. Additionally, patient anatomy‐based QA systems could replace independent monitor units (MU) checks in the patient geometry.

Only a limited number of measurement‐based 3D anatomy dose QA devices are clinically available: The 3DVH (Sun Nuclear Corporation, Melbourne, FL), the ${\text{MatriXX}}^{\text{Evolution}}$ (IBA Dosimetry, Schwarzenbruck, Germany), Delta^4DVH^ Anatomy (ScandiDos, Uppsala, Sweden). The measurement for Delta^4DVH^ Anatomy and the 3DVH system is performed in a stationary phantom, while the measurement device for the ${\text{MatriXX}}^{\text{Evolution}}$ is mounted on the gantry head and rotates together with the gantry.^(6)^ The Delta^4DVH^ Anatomy and the ${\text{MatriXX}}^{\text{Evolution}}$ use independent dose calculation algorithms, which calculate the dose to the patient using the energy fluency. The 3DVH does not recalculate the dose; instead it perturbs the TPS patient planned dose to account for known errors measured in the conventional QA.^7^, ^8^

This paper describes the evaluation of a new commercially available anatomy‐based QA system. The isocenter dose in a homogeneous phantom was measured to investigate the accuracy of the algorithm for static fields. The dose distribution was measured in the isocenter plane of two different homogeneous phantoms to compare the measured dose with the dose calculated by patient anatomy‐based QA algorithm for VMAT fields. To investigate the error detection capability of the system, an error‐induced treatment plan was created. Furthermore, a patient study was performed with ten plans for prostate cancer and ten plans for intracranial lesions.

### A. Treatment planning

Treatment planning was performed in Eclipse (Varian Medical Systems, Palo Alto, CA) treatment planning system (TPS). Dose calculation was done using the anisotropic analytical algorithm (AAA 8.9)^(9)^ with a calculation grid size of 2.5 mm. Sixteen open static fields were planned for the homogeneous OCTAVIUS (PTW, Freiburg, Germany) phantom with field sizes of $2\times 2\;{\text{cm}}^{2},5\times 5\;{\text{cm}}^{2},10\times 10\;{\text{cm}}^{2}$, and $20\times 20\;{\text{cm}}^{2}$ using four different energies (6 MV (X6), 10 MV (X10), X6 flattening filter free (X6FFF), and X10 flattening filter‐free (X10FFF)). Additionally, 16 VMAT fields were planned on two homogenous phantoms (OCTAVIUS and solid slab water‐equivalent phantom), simulating prostate treatments. For each phantom, one plan with two fields per energy was created. The progressive resolution optimizer (PRO 8.9) was used. A dose of 2 Gy per fraction was prescribed to the prostate PTV. Four OARs were contoured (rectum, bladder, and bilateral heads of femur) on the two phantoms and considered during optimization. Additionally, for ten nonconsecutive prostate cancer patients and ten patients with large intracranial lesions, treatment plans were performed prescribing 2−5 Gy per fraction to the PTV. All prostate patients were planned with a rectal balloon to protect the posterior part of the rectum.

### A.1 Introduced gantry positional error

A gantry positional error was introduced into the treatment plan to test the ability of the system to evaluate treatment delivery errors. An in‐house developed software program (in MATLAB, The MathWorks, Natick, MA) manipulates the treatment plan header of the exported DICOM plan file by overwriting the angle at each control point with the error‐introduced gantry angle. The gravitational response of the error is simulated with a sinusoid function to maximize the displacement when the gantry is perpendicular to the gravitational force (gantry at 90° and 270°) and to have a zero displacement at 0° and 180°.

### B. Treatment delivery and dose measurements

#### B.1 Linear accelerator

All measurements were performed at the University Hospital Zurich (U SZ) using the TrueBeam (Varian Medical Systems) linear accelerator. The energy modes X6, X10, X6FFF and X10FFF were used. The linear accelerator is calibrated such that 100 MU corresponds to 1 Gy at a depth of maximum dose for a field size of $10\times 10\;{\text{cm}}^{2}$ at a source surface distance (SSD) of 100 cm. The linear accelerator is equipped with a high‐definition multileaf collimator system (HDMLC). The HDMLC has 60 leaf pairs with a total length of 22 cm. The inner 8 cm are covered by 32 leaf pairs with a width of 0.25 cm. The outer 28 leaf pairs have a width of 0.5 cm. The dosimetric properties are described in more detail by Hrbacek et al.^(10)^

#### B.2 Delta^4PT^phantom measurements

Dose measurements were performed using the Delta^4PT^ (ScandiDos) phantom. Delta^4PT^ is a cylindrical phantom for pretreatment patient QA. Absolute dose is measured in 1069 p‐Si diode detectors arranged in two orthogonal detector arrays. The space between the detectors is 5 mm in the central section of the array $\left(8\times 8\;{\text{cm}}^{2}\right)$ and 10 mm outside $\left(20\times 20\;{\text{cm}}^{2}\right)$. The diameter of the detectors is 1 mm. Properties of the phantom have been described in detail by Bedford et al.^(11)^ and Sadagopan et al.^(2)^

#### B.3 Ion chamber measurements for static fields

The OCTAVIUS phantom has an orthogonal shape. It is 32 cm wide and has a length of 32 cm. Three water‐equivalent solid slabs of $31\times 10\times 2.2\;{\text{cm}}^{3}$ are inserted in the $30\times 30\times 2.2\;{\text{cm}}^{3}$ central cavity to allow the measurement with GAFCHROMIC film (GF) or PinPoint (PTW‐Freiburg) chamber (PC). The phantom is described in detail by Van Esch et al.^(12)^ Isocenter dose in the OCTAVIUS phantom was measured using the PC. The chamber model 31016 with an inner diameter of 2.9 mm shows a flat angular response, as the measuring volume is approximately spherical.

#### B.4 GAFCHROMIC film measurements for VMAT fields

The dose distributions in the frontal isocenter plane were measured in the two phantom patients using GF EBT2 (ISP, Wayne, NJ and Ashland Inc., Covington KY). For each sheet of film, an individual five step calibration was performed by irradiating five $5\times 3.2\;{\text{cm}}^{2}$ pieces of film, from 0 up to 300 MU in 75 MU steps with the X6 beam.

The films were scanned with a resolution of 150 dpi, resulting in a distance between two measurement points of 0.17 mm. The red channel transmission values were used for dose conversion and the blue channel values for film thickness correction.

The GF was placed on the top of the solid slabs inserted in the OCTAVIUS phantom and the VMAT plans were irradiated.

The water‐equivalent RW3 slab phantom (PTW) was constructed with 20 10 mm slabs with a diameter of $30\times 30\;{\text{cm}}^{2}$.^(13)^ The GF was placed in the central horizontal plane of the $20\times 30\times 30\;{\text{cm}}^{3}$ water phantom, where the dose distributions of the VMAT plans were measured. In a prestudy, we've evaluated the accuracy of the film dosimetry set up in our department and found a dose uncertainty of 1.8%.

### C. Anatomy dose calculation algorithm

The Anatomy dose (3D dose to patient) algorithm calculates the delivered dose to the patient's anatomy, based on the patient's CT. For each control point in a VMAT beam, the patient dose calculation algorithm converts the in the Delta^4PT^ measured detector doses in two steps.^(14)^ First, out of the measured dose in the detector array, the energy fluence is approximated using a pencil beam algorithm. Then the energy fluence is applied to the inhomogeneous patient geometry and the dose is calculated using the same pencil beam algorithm.

The pencil beam algorithm in the Delta^4DVH^ Anatomy option is commissioned using the three‐dimensional dose distributions of open squared fields ($3\times 3\;{\text{cm}}^{2}$ to $40\times 40\;{\text{cm}}^{2}$), exported from the treatment planning system. Additionally, headscatter factors for the same field sizes are needed.

The Delta^4DVH^ Anatomy option allows the comparison of the back‐projected measured patient anatomy dose with the TPS planned patient dose. Dose distributions can be compared based on DVHs or GAI. This GAI is calculated separately for each OAR, PTV, and body structure. The calculation grid size of the anatomy dose calculation algorithm is the same as in the TPS.

### D. Analysis of the measurements

#### D.1 Static field evaluation

The isocenter dose in the TPS and the Anatomy option in the two phantoms were determined and additionally the dose was measured. Relative dose differences were calculated. Mean and standard deviation were determined over the four measured open fields of $2\times 2\;{\text{cm}}^{2},5\times 5\;{\text{cm}}^{2},10\times 10\;{\text{cm}}^{2}$, and $20\times 20\;{\text{cm}}^{2}$ for each energy (X6, X10, X6FFF, X10FFF).

#### D.2 VMAT phantom plan evaluation

**Figure 2:**
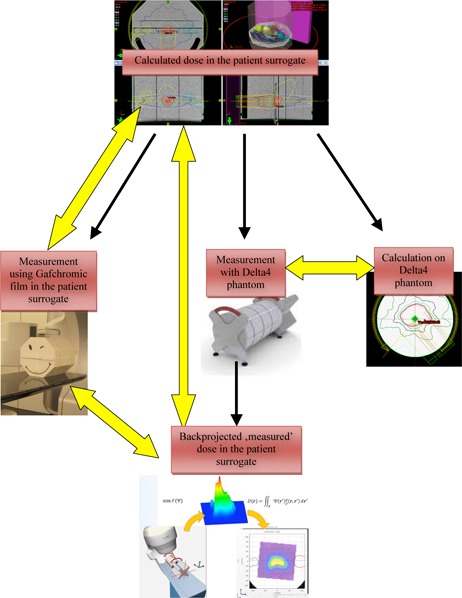
Evaluation of the Anatomy option using two phantoms as patient surrogates. A plan is calculated on a phantom and a verification plan is done on the Delta^4^ phantom. The plan is irradiated on the Delta^4^ phantom and on the patient surrogate phantom with a GAFCHROMIC film inserted. From the Delta^4^ measurements, the dose is back‐projected to the patient anatomy. The different calculated and measured dose distributions are compared (indicated with yellow arrows in the figure).

The dose distributions obtained during the measurements described above were compared (Fig. 2). Four different comparisons were made, all resulting in a GAI:

1. The calculated dose distribution exported from the TPS was compared to the back‐projected dose distribution calculated by the Delta^4^ Anatomy software in three dimensions (Anatomy GAI)
2. The calculated dose distribution in the isocenter plane exported from the TPS was compared to the GF measurement (TPS‐Phantom GAI)
3. The back‐projected dose distribution in the isocenter plane calculated by the Delat^4^ Anatomy software was compared to the measured GF dose distribution (Anatomy‐Phantom GAI),
4. The Delta^4^ measurement was compared to the dose calculated on the Delta^4^ phantom (Conventional GAI).

#### D.3 VMAT patient plan evaluation

Ten treatment plans for prostate cancer treatment and ten plans for intracranial lesions with 2‐3 VMAT fields were investigated. The planned dose distributions for the patients were exported from the TPS to the ScandiDos QA software. The plans were measured on the Delta^4PT^ phantom and the dose was back‐projected onto the patient in the Delta^4DVH^ Anatomy software. The conventional GAI and a PTV and body structure GAI were calculated.

#### D.4 Gamma analysis and statistics

For the phantom study, the dose planes were evaluated using a GAI.^(1)^ As gamma criteria, 1 up to 3 mm DTA combined with 1% up to 3% DD (from isocenter dose, which was for all plans in the high‐dose region) were used. For the VMAT patient plan evaluation, a DD of 3% and 5% and a DTA of 3 mm were used. Voxels with less than 20% of the isocenter dose were not taken into account. This ensured that the analysis was focused on the clinically relevant region. The calculated dose was used as reference.

The two VMAT fields per plan were measured separately and evaluated together. Mean and standard deviation (SD) of dose difference, GAI, and GAI‐change were calculated. All values in the result part are given as mean±SD.

*D.5 Introduced error plan evaluation*

The modified plans were radiated on the treatment machine. This allowed a comparison of the measured error‐introduced dose distributions with the calculated error‐free dose distribution. Finally, the relative Anatomy‐ and Conventional‐GAI change to the error‐free GAIs was calculated.

## III. RESULTS

### A. Open fields in the homogeneous phantom

The dose deviation of the anatomy dose at the isocenter compared to the measurements with the PC was on average over the four fields and four energies 1.1%±0.6%. The TPS isocenter dose compared with the PC measurement showed a mean deviation of 0.6%±0.4% and the anatomy versus the TPS isocenter dose showed a mean deviation of 0.7%±0.5%. Table 1 summarizes the dose deviations for the different field sizes.

**Table 1:** Comparison of the mean dose measured in the isocenter using a pinpoint chamber with the dose calculated by the Anatomy option and by the Eclipse treatment planning system (TPS)

	$2\times 2\;cm^{2}$	$5\times 5\;cm^{2}$	$10\times 10\;cm^{2}$	$20\times 20\;cm^{2}$
Anatomy vs. Pinpoint Chamber	1.5%±0.5%	1.3%±0.2%	0.9%±0.9%	0.7%±0.2%
TPS vs. Pinpoint Chamber	0.9%±0.5%	0.8%±0.4%	0.4%±0.3%	0.3%±0.2%
Anatomy vs. TPS	0.6%±0.4%	0.6%±0.4%	1.0%±0.7%	0.7%±0.4%

### B. VMAT fields in the homogeneous phantoms

All measured VMAT plans had a Conventional GAI of 100% for the passing criteria of 3 mm/3%. The GAI dropped to a mean value of 88.1%±7.3% for 1m/1%.

**Figure 3:**
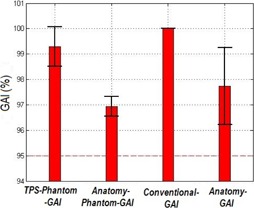
GAI (3 mm/3%) for the four comparisons as a mean value for both phantoms and the four energies used. Error bars are the standard deviation.

**Figure 4:**
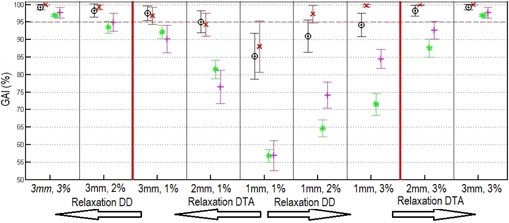
GAI for the four comparisons, as an average of both phantoms and the four energies. The red × = the Conventional‐GAI, the black circles = the TPS Phantom GAI, the green stars = the Anatomy Phantom GAI, and the pink crosses = the Anatomy GAI for the different DTA and DD. From the center to the right, the big red line is a relaxation of the DD and to the left, the red line is an relaxation of the DTA. Error bars are the standard deviation.

The mean value of the OCTAVIUS TPS Phantom GAI (3 mm/3%) for the four energies was 98.6%. The OCTAVIUS Anatomy Phantom GAI gave an average of 95.4% (3 mm/3%). For the water phantom, the average TPS Phantom GAI (3 mm/3%) was 100.0%. The Anatomy Phantom GAI (3 mm/3%) had an average of 98.5%. Concerning all energies and both phantoms, the mean Anatomy Phantom GAI (3 mm/3%) was 96.9%±0.4%. The average of the TPS Phantom GAI (3 mm/3%) was 99.3%±0.8%, and the Anatomy GAI (3 mm/3%) showed an average of 97.7%±1.5%. The Anatomy GAI had the biggest standard deviation out of the four comparisons. In Fig. 3, the GAIs (3 mm/3%) with the standard deviation for the four different comparisons are shown, and in Fig. 4 the GAIs for the different DTA and DD constraints are presented. According to our internal guidelines for patient specific VMAT QA, plans are accepted if the QA GAI is above 95% (3 mm/3%).

### C. VMAT patient plan evaluation

All plans of the prostate cancer patients used to investigate the conversion algorithm passed the conventional ScandiDos QA test with 99.8%±0.4% Conventional GAI (3%/3 mm). The plans of the intracranial lesion passed with a mean GAI of 99.7%±0.4%.

For DTA=3 mm and DD=3%, the Anatomy GAI was 86.0%±5.0% (prostate) and 95.3%±2.1% (intracranial lesions). The GAI for the PTV was 83.7%±8.8% and 90.6%±2.3% for prostate and intracranial lesions, respectively. For DTA=3 mm and DD=5%, the PTV‐GAI were between 93.1% and 99.6%, and the Anatomy GAI between 90.1% and 99.2%. All results are shown in Table 2.

**Table 2:** Mean three‐dimensional gamma agreement index (GAI) and standard deviation inside the PTV and the body of the patient using Delat^4^ Anatomy option. Mean GAI in the horizontal isocenter slice and mean dose deviation in the isocenter and mean GAI in the phantom (Conventional GAI)

	*Prostate*	*Brain*
Body GAI (3 mm/5%)	94.0%±2.9%	97.9%±1.5%
PTV GAI (3 mm/5%)	97.3%±2.0%	94.4%±1.7%
Body GAI (3 mm/3%)	86.0%±5.0%	95.3%±2.1%
PTV GAI (3 mm/3%)	83.7%±8.8%	90.6%±2.3%
Horizontal slice (3 mm/3%)	87.2%±5.1%	95.2%±2.3%
Dose deviation (%)	2.4%±2.5%	1.3%±2.1%
Conventional GAI (3 mm/3%)	99.8%±0.4%	99.7%±0.4%

### D. Introduced errors

**Figure 5:**
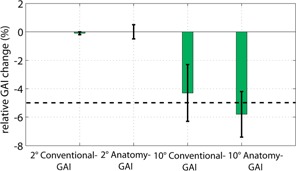
The relative GAI (3 mm/3%) change for a 2° and 10° gantry angle error. Error bars are the standard deviation.

Figure 5 shows the changes in the GAI index if a gantry positional error is introduced. For DTA=3 mm and DD=3% and an angle error of 2°, the relative GAI change for Conventional GAI was −0.1%±0.1% considering all energies and both phantoms. The mean change in Anatomy GAI was 0.0%±0.5%. For a 10° angle error, the change in the Conventional GAI was −4.3%±2.0% and in the Anatomy GAI the change was −5.8%±1.6%.

In the case of an angle error of 2°, the mean Conventional GAI change over all energies and both phantoms with DTA=1 mm and DD=1% was −5.4%±1.2%. The mean change for the Anatomy GAI was −0.8%±1.7%. For an error of 10°, the Conventional GAI change was −25.2%±3.6% and for the Anatomy GAI, −19.9%±2.9%.

## IV. DISCUSSION

Recently, 3D anatomy‐based QA systems have gained popularity.^6^, ^7^, ^8^, ^15^, ^16^, ^17^ A more precise idea of the dose distribution within the patient becomes increasingly important with more sophisticated treatment plans. Anatomy‐based QA allows access to structure specific GAI and DVHs (Fig. 1). This induces discussions about the clinical relevance of deviations between the measured and the TPS‐calculated dose distribution within the patient. Radiation plan adjustments can then be made before the treatments start.

In this study, the Delta^4DVH^ Anatomy 3D QA system was evaluated. With two homogeneous phantoms the conversion algorithm was confirmed. Angular errors with a gravitational response up to 10° were introduced to investigate the relative change in the Anatomy GAI and the Conventional GAI. It was shown that the Delta^4DVH^ Anatomy 3D QA detects the error as good as the conventional method. Furthermore, Delta^4DVH^ Anatomy conversions for 20 patients resulted in a PTV‐GAI (3 mm/5%) of 97%. However, it dropped to 84% for the passing criteria 3 mm/3%. All patient plans passed the conventional QA test (Conventional GAI>%,3 mm/3%).

The anatomy dose conversion algorithm calculated the isocenter dose in a homogeneous phantom accurately for static open fields of $2\times 2\;{\text{cm}}^{2}$ up to $20\times 20\;{\text{cm}}^{2}$ and energies of X6, X10, X6FFF, and X10FFF. It was shown that the TPS predicts the isocenter dose slightly better than the back‐projected Anatomy dose.

The mean Anatomy Phantom GAI (3 mm/3%) showed that the anatomy dose (3D dose to patient) algorithm works for a homogeneous case. While all the Conventional GAI had 100.0%, the mean Anatomy GAI was 98%. Lowering the passing criteria to DTA=1 mm and DD=1% resulted in a 28% lower GAI for the two comparisons with the back‐projected Anatomy dose (Anatomy GAI and Anatomy Phantom GAI) than the two comparisons with the calculated TPS dose (TPS Phantom GAI and Conventional GAI) (Fig. 4). This may result from the different dose calculation algorithms used. Eclipse TPS uses the AAA algorithm, which is a more advanced algorithm than pencil beam that is used in the Delta^4DVH^ Anatomy software. ^(18)^ Another cause for the lower agreement may be that the diode grid resolution is too small to accurately determine the energy fluence used for dose calculation in the patient.

The error detection of the Delta^4DVH^ Anatomy was as good as the conventional measurement. A gravitational gantry error up to 2° was not detectable, which was to be expected, as small random and systematic gantry position errors are relatively insignificant.^(19)^ For the investigated two phantoms at a gantry error of 10°, six out of eight incorrect plans passed the conventional ScandiDos QA test (Conventional GAI>95%, relative Conventional GAI change <5%). With the Delta^4DVH^ Anatomy, just two out of eight incorrect plans passed (Relative Anatomy GAI change <5%). However, the problem is the large variation of the Anatomy GAI in the error‐free case. One of the eight plans did not pass the Anatomy QA conditions (Anatomy GAI>95%,3 mm/3%), even with the error‐free measurement, while all of them passed the standard ScandiDos QA test with 100% Conventional GAI. Therefore, it is difficult to judge the Anatomy QA results.

The constraints for the DD had to be loosened from 3% to 5% for real patient geometries. This is most likely due to inaccurate dose calculation using the pencil beam algorithm in the Delta^4DVH^ Anatomy.^(20)^ This is especially a problem for the prostate dose distributions, because the PTV includes parts of the air‐filled rectal balloon. The average overall body GAI and PTV‐GAI rose by about 10% when changing the DD passing criteria from 3% to 5%.

A similar investigation was done for the ${\text{MatriXX}}^{\text{Evolution}}$ system, showing a dose deviation of the TPS of less than 3%, compared to the reconstructed dose in the patient.^(15)^ The more accurate results for the ${\text{MatriXX}}^{\text{Evolution}}$ system compared to the Delta^4DVH^ Anatomy can be explained by the different dose conversion algorithms. ${\text{MatriXX}}^{\text{Evolution}}$ uses a collapsed cone convolution algorithm,^(21)^ which is in inhomogeneous media, superior to the pencil beam algorithm.

The 3DVH system was evaluated in a white paper by Sun Nuclear.^(22)^ The authors created a plan and introduced errors into the plan. It was then irradiated on the conventional QA phantom and subsequently converted to dose in the patient. The conversion was done by perturbing the error‐free dose distribution in the patient with the measured errors. The dose distributions with introduced errors from the planning system were compared with the ‘measured’ dose distribution in the patient. The plan error was quantified with the GAI (2 mm/2%) of the conventional QA (error‐induced versus measured error‐free plans). The perturbation algorithm converted even erroneous dose distributions, which had 12% conventional GAI, back to 96% anatomy GAI (error‐free versus perturbation‐corrected dose distributions). The authors reported dose deviations in the range of −0.31% to 2.08%, which is similar to our results. However, the methods used are quite different and make a quantitative comparison difficult.

## V. CONCLUSIONS

The anatomy‐based, patient‐specific quality assurance system from ScandiDos (Delta^4DVH^ Anatomy), evaluated with two phantoms, predicts the dose distribution correctly for a homogeneous case. The large variation of the GAI in the ‘error‐free plans’ is a limiting factor when it comes to error detection. The constraints for the DD had to be loosened from 3% to 5% for real patient geometries, to achieve an acceptable GAI. The dose calculation algorithm is inferior to that used in the TPS (Eclipse). This inaccuracy is assumed to be due to the pencil beam algorithm of the Delta^4DVH^ Anatomy.

## Supporting information

Supplementary Material
