# Supplementary figures and images for: Clinical evaluation of an anatomy‐based patient specific quality assurance system
Source: J Appl Clin Med Phys. 2014 Mar 6;15(2):181–90. doi: 10.1120/jacmp.v15i2.4647 (PMC5875461; doi:10.1120/jacmp.v15i2.4647)

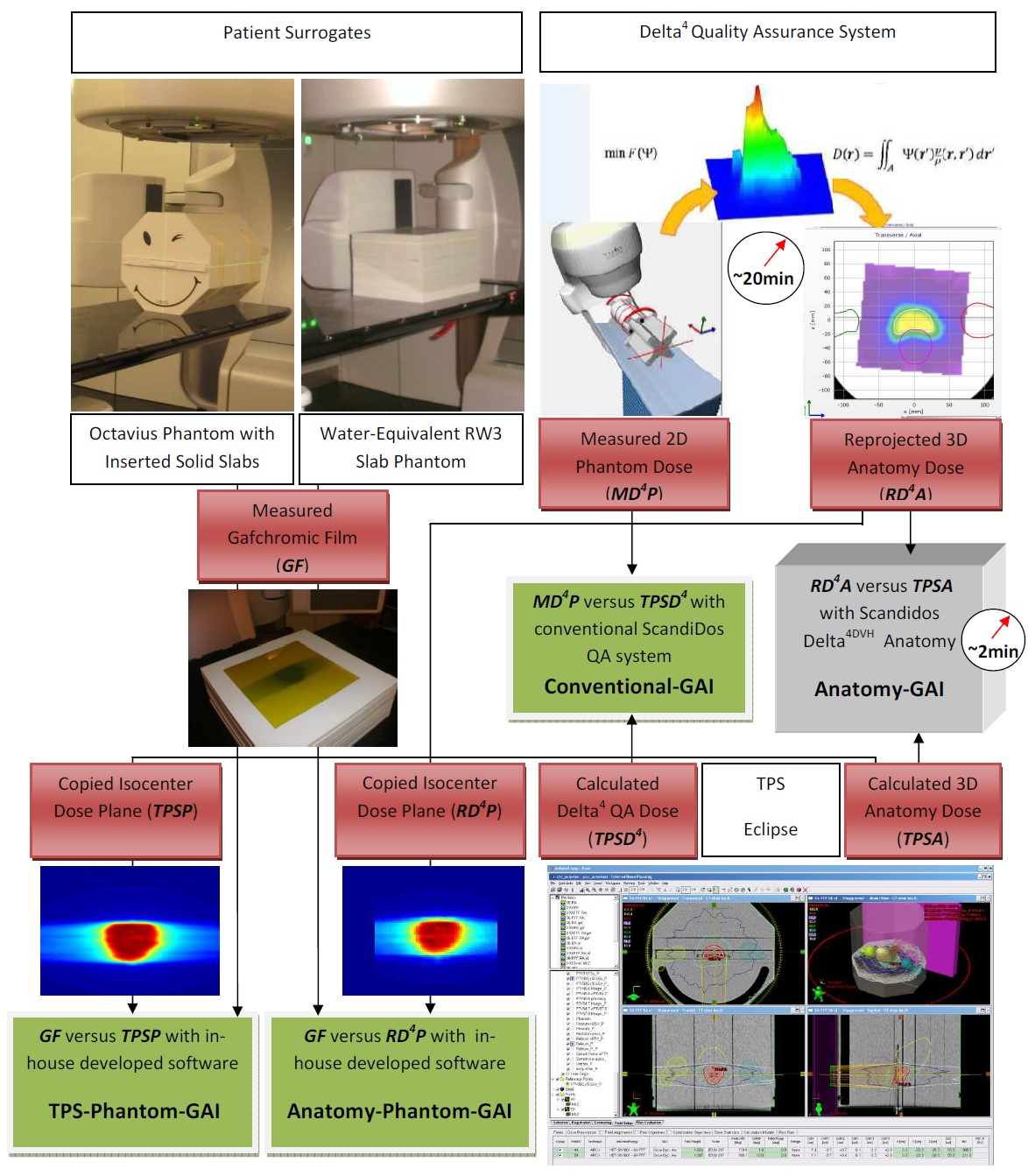

Supplement: Supplementary file 1 — Supplementary Material [file ACM2-15-181-s001.jpg]
